# Changes in Skeletal Muscle and Body Weight on Sleeping Beauty Transposon-Mediated Transgenic Mice Overexpressing Pig mIGF-1

**DOI:** 10.1007/s10528-018-9848-7

**Published:** 2018-02-22

**Authors:** Bo Gao, Wei Wang, Han Wu, Cai Chen, Dan Shen, Saisai Wang, Wei Chen, Li Zhang, Shuheng Chan, Chengyi Song

**Affiliations:** Joint International Research Laboratory of Agriculture and Agri-product Safety, College of Animal Science & Technology, Yangzhou University, Yangzhou, 225009 China

**Keywords:** Pig insulin-like growth factor 1, Transgenic mice, Skeletal α-actin gene regulator, Sleeping Beauty transposon

## Abstract

Insulin-like growth factor (IGF-I) is an important growth factor in mammals, but the functions of the local muscle-specific isoform of insulin-like growth factor 1 (mIGF-1) to skeletal muscle development have rarely been reported. To determine the effect of pig mIGF-1 on body development and muscle deposition in vivo and to investigate the molecular mechanisms, the transgenic mouse model was generated which can also provide experimental data for making transgenic pigs with pig endogenous IGF1 gene. We constructed a skeletal muscle-specific expression vector using 5′- and 3′-regulatory regions of porcine skeletal α-actin gene. The expression cassette was flanked with Sleeping Beauty transposon (SB)-inverted terminal repeats. The recombinant vector could strongly drive enhanced green fluorescence protein (EGFP) reporter gene expression specifically in mouse myoblast cells and porcine fetal fibroblast cells, but not in porcine kidney cells. The EGFP level driven by α-actin regulators was significantly stronger than that driven by cytomegalovirus promoters. These results indicated that the cloned α-actin regulators could effectively drive specific expression of foreign genes in myoblasts, and the skeletal muscle-specific expression vector mediated with SB transposon was successfully constructed. To validate the effect of pig mIGF-1 on skeletal muscle growth, transgenic mice were generated by pronuclear microinjection of SB-mediated mIGF-1 skeletal expression vector and SB transposase-expressing plasmid. The transgene-positive rates of founder mice and the next-generation F1 mice were 30% (54/180) and 90.1% (64/71), respectively. The mIGF-1 gene could be expressed in skeletal muscle specifically. The levels of mRNA and protein in transgenic mice were 15 and 3.5 times higher, respectively, than in wild-type mice. The body weights of F1 transgenic mice were significantly heavier than wild-type mice from the age of 8 weeks onwards. The paraffin-embedded sections of gastrocnemius from 16-week-old transgenic male mice showed that the numbers of myofibers per unit were increased in comparison with those in the wild-type mice. mIGF-1 overexpression in mice skeletal muscle may promote myofibers hypertrophy and muscle production, and increased the average body weight of adult mice. Transgenic mice models can be generated by the mediation of SB transposon with high transgene efficiency.

## Introduction

Insulin-like growth factor 1 (IGF-1) plays a fundamental role in cell proliferation and differentiation, and regulates postnatal mammalian growth and development (Tonkin et al. 2015). The primary structure of IGF-1 is highly conserved in placental mammalian species. Canine, bovine, ovine, porcine, and human IGF-1 s are identical, whereas rat and mouse IGF-1s differ from human IGF-1 by 3 and 4 amino acids, respectively (Shavlakadze et al. 2005). The IGF-1 gene is a single copy and is highly conserved in structure in mammals, containing six exons and five introns. The heterogeneity of IGF-1 transcripts is complicated because of the use of alternative transcription start sites (Yang et al. 1995), alternative posttranscriptional exon splicing (Bell et al. 1986), and the use of different polyadenylation sites (Lund et al. 1989). IGF-1 mRNA was expressed in different tissues at different abundance. The predominant IGF-1 mRNA variant expressed in skeletal muscle (Sk muscle) is initiated at exon 1 (Class 1, C1) and represents an exon 4–6 spliced variant, which encodes an IGF-1 isoform containing an Ea peptide (Musaro et al. 2001), which is also referred to as a “local muscle-specific” isoform of IGF-1 (mIGF-1). Overexpression of human or mouse mIGF-1 can significantly promote muscle growth of transgenic (Tg) mice (Coleman et al. 1995; Musaro et al. 2001). mIGF-1 can promote myoblast proliferation and fusion of myofibers, which can result in muscle hypertrophy (Coleman et al. 1995). mIGF-1 knock-out mice showed slow growth and immature or early death, with serious defects in muscle development (Shavlakadze et al. 2005).

The effects of promoters on transgene expression are important. Coleman et al. (1995) utilized the metallothionein promoter to drive expression of human mIGF-1 cDNA in Tg mice, resulting in IGF-1 overexpression in a broad range of visceral internal organs and increased concentrations of IGF-1 in serum. These Tg mice exhibited an increase in body weight and organomegaly, but only a modest improvement in muscle mass. To test the effects of mIGF-1 overexpression on Sk muscle growth and physiology, it would be necessary to target its overexpression specifically in Sk muscle. At present, two promoters of skeletal α-actin (Asante et al. 1994) and myosin light chain (MLC1/3) (Rosenthal et al. 1989) are often used, which are active in differentiated skeletal or cardiac muscle cells. Skeletal α-actin is the major actin isoform in adult Sk muscle (Gunning et al. 1987; Asante et al. 1994), which suggests that the promoter for skeletal α-actin is strong. The contiguous 3′-untranslated region (UTR) of the skeletal α-actin gene can directly correct the temporal and spatial expression of skeletal actin-based transgenes in mice (Brennan and Hardeman 1993).

The Sleeping Beauty (SB) transposon system is extremely effective at delivering DNA to vertebrate genomes (Ivics et al. 2004). The system consists of two parts: a transposon and a source of transposase. The transposon vector consists of inverted terminal repeats (ITRs) that flank an expression cassette. The transposon can be mobilized when SB transposase is supplied in transgene. Transposase binds at precise sites in each of the ITRs where it cuts out the transposon and inserts it into a new DNA locus (Geurts et al. 2003). SB-mediated transgene delivery is well developed in fish, mammals (mouse, rabbit, and pig), frogs, and poultry (Carlson et al. 2011a; Mátés 2011; Ivics et al. 2014; Tschida et al. 2014). As an efficient transgene delivery tool, the SB transposon system has four attractive features: low immunoreactivity, easy uptake of transposons into cell nuclei by the nuclear localization signal of SB transposase (Zanta et al. 1999), large carrying capacity of the transposon for efficient integration into chromosomes, and long-term expression stability of transposed genes even following passage through the germ-line (Luo et al. 1998; Yant et al. 2000; Fischer et al. 2001; Horie et al. 2001; Carlson et al. 2011b).

In this report, a myogenic expression vector containing regulatory elements from both the 5′- and 3′-flanking regions of the pig skeletal α-actin gene was constructed, and a transgenic mouse model expressing pig mIGF-1 specifically in Sk muscle was generated. To increase the transgene efficiency, the SB transposon was used to mediate the myogenic expression vector. This paper studied the effect of pmIGF-1 overexpression in Sk muscle on Tg mice growth and the proliferation and differentiation of myoblasts, and provides an effective method for generating transgenic pigs.

## Materials and Methods

### Construction and Characterization of Skeletal Muscle-Specific Expression Vector

**Fig. 1:**
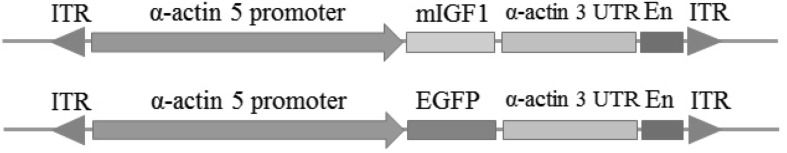
Schematic diagram of skeletal-specific expression vector mediated by Sleeping Beauty (SB) transposon. ITR: inverted terminal repeat of SB; α-actin 5 promoter: 5′-regulator for skeletal α-actin gene; mIGF-1: mIGF-1 open reading frame; α-actin 3 UTR: 3′-untranslated region and contiguous 3′-noncoding region for skeletal α-actin gene; En: SV40 enhancer

SB transposon-mediated vectors were constructed using the standard molecular cloning techniques. To construct skeletal-specific expression vectors, pig skeletal α-actin gene regulators including the 5′ core promoter, upstream activating sequences, natural cap site, 5′-UTR (exon 1), first intron, and portions of exon 2 up to the initiation ATG sequence. 3′-UTR and contiguous 3′-noncoding regions were amplified from pig genome DNA by high-fidelity polymerase chain reaction (PCR) using primers designed according to sequences published previously (Table 1). The PCR products were cloned into pGEM-T vectors and confirmed by sequence analysis. The 5′- and 3′-regulatory regions were then subcloned into the pGL3-control vector and the resulting vector was named pGL3-2. mIGF-1 cDNA was cloned from pig liver total RNA by reverse-transcription PCR using primers designed according to sequences published previously (Table 1). After sequencing, the mIGF-1 cDNA was subcloned downstream of the 5′-regulatory region of pGL3-2 into the *Eco*RI and *Nco*I restriction enzyme sites, and the resulting vector was named pA-actin-mIGF-1. Then, the mIGF-1 expression cassette was cut from pA-actin-mIGF-1 with *Eco*RV and subcloned into *Eco*RV sites in the SB transposon vector pT2-HB (provided by Dr. Moore, University of Minnesota). The resulting vector was named pT2/A-actin-mIGF-1 (Fig. 1).

**Table 1:** Primer sequences for vector construction

	Sequences (5′→3′)	Product (bp)
α-actin-5	F: cg*GGTACCGATATC*cagcagaaattgacggaaca	2512
	R: aa*ACGCGT*at*GAATTC*ggcgtcgggtttctgcaa	
α-actin-3	F: aat*CCATGG*accccattccaacagctg	751
	R: ag*GTTAAC*ta*GCGGCCGC*atcaacacccggcttgaa	
SV40 enhancer	F: at*GCGGCCGC*tgaacgatggagcggaga	242
	R: gc*GTTAACGATATC*cgctgtggaatgtgtgtca	
mIGF-1	F: gc*GAATTC*ttgcacttcagaagcaatgg	478
	R: gc*CCATGG*ctacattctgtagttcttgtttcc	
eGFP	F: atGAATTCatggtgagcaagggcgagga	720
	R: agCCATGGttacttgtacagctcgtcca

To evaluate whether the vector could specifically drive expression of the gene of interest in skeletal cells, IGF-1 was replaced by the enhanced green fluorescence protein (EGFP) reporter gene and the recombinant vector pT2/A-actin-EGFP (Fig. 1) was transfected into mouse myoblast cells (C2C12; ATCC CRL-1772), porcine fetal fibroblast cells (PEF; primary culture), and porcine kidney cells (PK15; ATCC CCL-33). One day before transfection, 2 × 10^5^ cells per well were plated in a 24-well plate. When the cells were 90–95% confluent, 0.8 μg DNA packed with 2 μL Lipofectamine 2000 was added according to the manufacturer’s instructions (Invitrogen, Carlsbad, CA, USA) to each well. The cells were incubated at 37 °C in a CO_2_ incubator for 24 h prior to testing for transgene expression. For each cell type, three treatment groups [pT2/A-actin-EGFP, pEGFP-N1, and blank (Lipofectamine 2000 only)] were tested, with each group tested in triplicate.

### Generation, Detection, and Breeding of Transgenic Mice

The SB transposon plasmid pT2/A-actin-mIGF-1 and transposase-encoding plasmid pCMV-SB11 (provided by Dr. Moore, University of Minnesota) were prepared using an EndoFree plasmid maxi preparation kit (Qiagen, German). Transposon DNA and transposase DNA were mixed at the mass ratio of 2:1 and microinjected into the cytoplasm of C57BL/J6 mice fertilized eggs. G_0_ founder mice were tested for transgene integration using PCR and Southern blot analysis. Mouse tail DNA was isolated and subjected to PCR using specific primers (Table 2). The length of target products was 545 bp. Southern blot analysis was used to identify PCR-positive founder mice. Tail genomic DNA was digested with the restriction enzyme *Eco*RV, separated on a 0.8% (w/v) agarose gel, and transferred to positively charged nylon membranes (Roche). The blots were hybridized with 311 bp DIG-labeled probes prepared with the PCR DIG probe synthesis kit (Roche) using the primers designed according to the sequence of mIGF-1 cDNA (Table 2). The fragment of pT2/A-actin-mIGF-1 plasmid DNA digested with *Eco*RV was used as a positive control. The transgenic mouse experiments were carried out in the Institute of Zoology, Chinese Academy of Sciences. The three male founders were mated with wild-type (Wt) female mice at the ratio of 1:4 to generate three transgenic lines. Wt mice lines were used as the control.

**Table 2:** Primers for screening transgenic mice

	Primer sequences (5′**→**3′)	Product (bp)
P1 (α-actin 5′)	F: ccaggttgctcggattgat	206
	R: attgggttggaagactgctg	
P2 (α-actin 3′)	F: atcgtggatgagtgctgctt	251
	R: cacttgagcagattcgtcgt	
P3 (pmIGF-1 cDNA)	F: gccttgctgatcttgcagaa	545
	R: gaagtcgcagctgttggaat	
Probe primer	F: cagcagtcttccaacccaat	311
	R: acatctccagcctcctcaga	

### Transcriptional Profiling of Pig mIGF-1 by Real-Time Quantitative PCR Assays

The total RNA of fresh tissues from transgenic and normal mice, including Sk muscle, heart, liver, lung, kidney, testis, and seminal vesicle, were isolated using TRIzol Reagent (Invitrogen). The first-strand cDNA was synthesized with 2 μg total RNA as template using RevertAid™ First-Strand cDNA Synthesis Kit (Thermo). The mIGF-1 expression level in each tissue was detected by qPCR (Applied Biosystems 7500 Real-Time PCR System) using SYBR Premix Ex Taq (Takara). Each assay was conducted with three tissue samples and the qPCR reactions for each sample were set up in triplicate. Endogenous mouse β-actin gene was used as an internal control. The specific primers are listed in Table 3. Identical amplification reaction conditions consisting of DNA denaturation at 95 °C for 30 s, 40 cycles each of the denaturation step at 95 °C for 5 s, and an annealing/extension step at 60 °C for 34 s were used for each gene analyzed. A final dissociation stage consisted of 95 °C for 15 s, 60 °C for 1 min, and 95 °C for 15 s. The reaction volume was kept at 20 μL by including 1 μL cDNA. The relative abundance of transcripts of each gene was calculated according to the comparative 2^−ΔΔCT^ method (Schefe et al. 2006).

**Table 3:** Primers for real-time quantitative PCR

	Primer sequences (5′**→**3′)	Product (bp)
IGF-1	F: TTATTTCAACAAGCCCACA	111
	R:TACATCTCCAGCCTCCTCA	
β-actin	F: CTCTTTTCCAGCCTTCCTT	112
	R: GTGTTGGCATAGAGGTCTT	

### Translational Profiling of Pig mIGF-1 by Western Blot

To prepare proteins from tissue samples, ~ 100 mg of fresh or frozen (− 80 °C) tissue samples were placed in an Eppendorf tube. Approximately 1 mL of ice-cold lysis buffer was rapidly added to the tube. The tissue was homogenized with an electric homogenizer, vortexed, and mixed well several times, and then centrifuged for 10 min at 12,000 rpm, 4 °C. The concentration of the protein in the supernatant was tested. Samples (40 μg) of total denatured protein from the tissue homogenate were boiled with 1 × loading buffer at 100 °C for 5 min and loaded into the wells of a 20% SDS-PAGE gel for separation. Proteins were transferred from the gel to a PVDF membrane (Millipore). The membrane was incubated with rabbit anti-human IGF-1 antibody at 1:200 dilution (Abcam) and goat anti-Rabbit IgG H&L (HRP) secondary antibody at 1:5000 dilution (Abcam). The signal was developed with the chemiluminescent HRP substrate ECL (Amersham), and images were acquired using darkroom development techniques for chemiluminescence. Mouse β-actin was used as an internal control.

### Phenotypic Analysis of Transgenic mice

The body weight of male or female Tg mice or normal mice was detected from 3 to 16 weeks of age. Growth curves based on body weight were used to compare the growth rate between transgenic and normal mice. Hind leg gastrocnemius obtained from 16-week-old male mice were fixed in 4% PFA for 24 h at room temperature, dehydrated with ethanol, and embedded in paraffin. Histopathologic evaluation was performed on deparaffinized sections stained by routine hematoxylin and eosin (H&E) staining.

### Statistical Analyses

Unpaired *t* tests were used for comparisons between age-matched control and pmIGF-1 Tg mice. Statistical significance was accepted for comparisons where *P* < 0.05.

## Results

### Characterization of the Transgene Construct in Tissue Culture

**Fig. 2:**
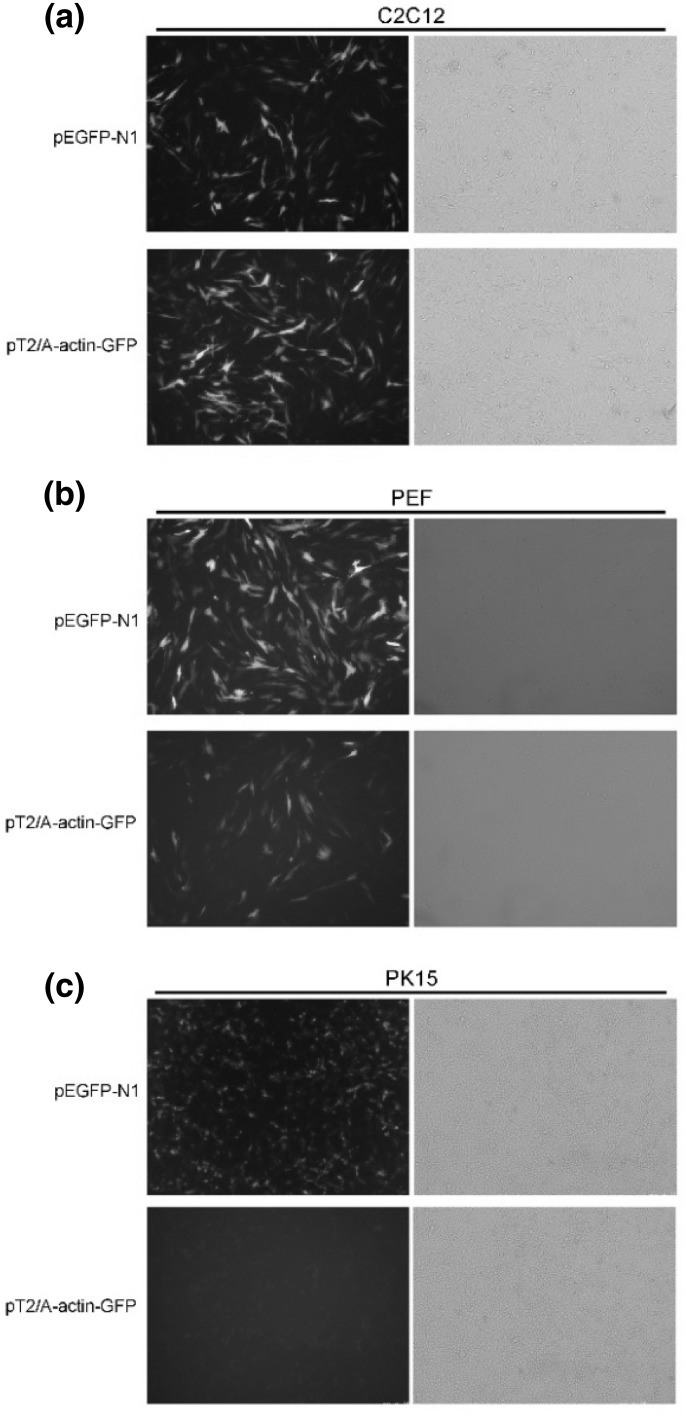
Green fluorescent protein expression in different cell types. The vector pT2/A-actin-EGFP was transfected into **a** mouse myoblast (C2C12), **b** porcine fetal fibroblast (PEF), and **c** porcine kidney (PK15) cells. pEGFP-N1 was used as a positive control

To validate the Sk muscle-specific expression vector, pT2/A-actin-EGFP was transfected into C2C12, PEF, and PK15 cells. After 24 h of transfection, green fluorescence could be tested under the fluorescence microscope. The results showed that EGFP was expressed in C2C12 and PEF cells, but not in PK15. The GFP fluorescence signals in C2C12 were significantly stronger than those in PEF, which suggested that the expression level of pT2/A-actin-EGFP in C2C12 was significantly higher than that of pEGFP-N1 (Fig. 2). These results suggested that the constructed vector was suitable for Sk muscle-specific expression, and that the 5′- and 3′-regulatory regions of α-actin were more favorable for effectively expressing exogenous genes than cytomegalovirus promoters in Sk muscle.

### Generation of Pig mIGF-1 Transgenic Mice

**Fig. 3:**
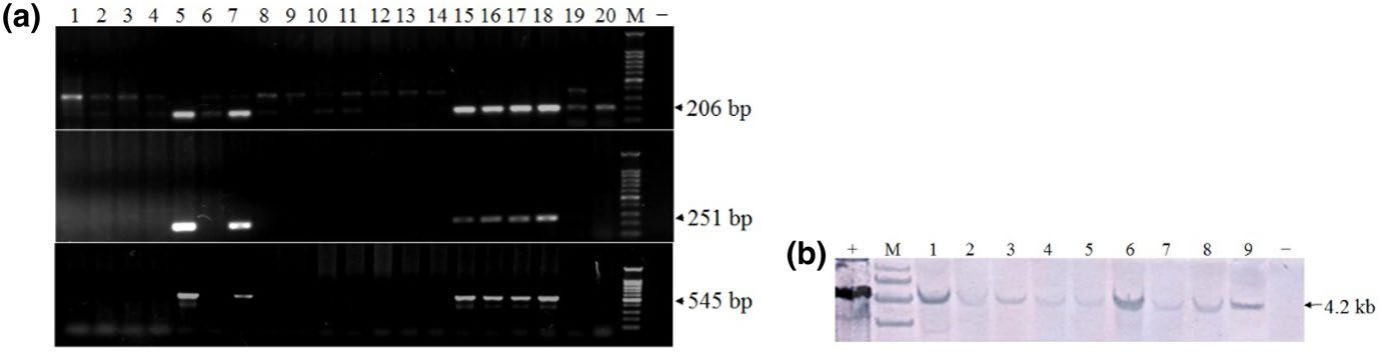
F_0_ founder mice were tested for transgene integration. **a** Mouse tail genomic DNA was subjected to PCR using three pairs of specific primers designed according to transgene sequences: α-actin-5′ (206 bp), α-actin-3′ (251 bp), pIGF-1-flank (545 bp). 1–20, F_0_ founders; M, 100 bp DNA ladder; -, Wt mouse; **b** PCR-positive individuals were tested by Southern blot analysis. Mouse tail genomic DNA was digested with *Eco*RV and hybridized with DIG-labeled probes. +, pT2/A-actin-IGF-1/*Eco*RV; M, DNA molecular weight marker III labeled with Digoxigenin; 1–9, PCR-positive F_0_ founders, -, Wt mouse

The pig mIGF-1 Sk muscle-specific expression cassette harbored by the SB transposon and SB transposase expression plasmid were co-microinjected into the male pronucleus of zygotes from the C57BL/J6 mice, which led to 180 live births. Based on the PCR and Southern blot analyses of tail DNA (Fig. 3), 54 founders were identified to be transgenic. The transgene efficiency was 30%, which was higher than that for common plasmid vectors (0.5–5%) (Tesson et al. 2005). There were three male mice (1#, 2# and 9#) among the 54 founders. The three male founders generated three transgenic lines by mating with Wt mice. The average positive rate of F1 was about 90.1% (Table 4).

**Table 4:** Summary results for transgenic mouse lines

Founder♂ X wild-type C57BL/J6♀	Total no. of pups	No. of IGF-1^+^ pups	Percentage of IGF-1^+^ pups
Line 1#	23	21	91.3
Line 2#	23	20	87.0
Line 9#	25	23	92.0
Total	71	64	Average 90.1

### pmIGF-1 Expression Level in Tissues of Transgenic Mice

**Fig. 4:**
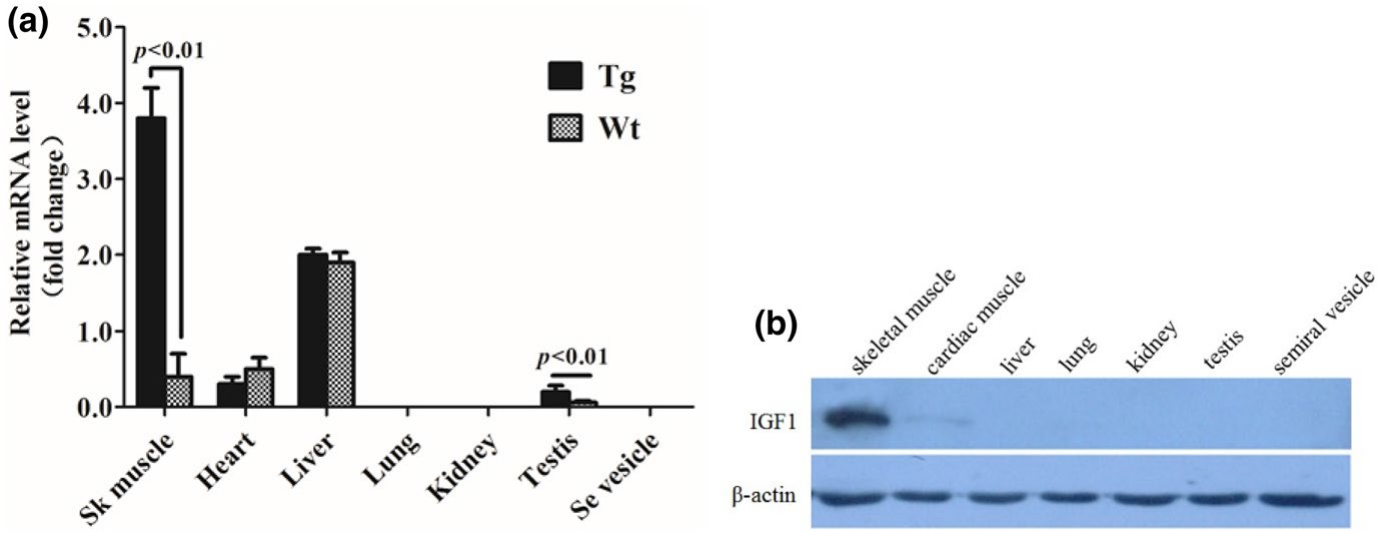
IGF-1 gene expression in transgenic mouse tissues. **a** IGF-1 mRNA expression levels determined by real-time quantitative PCR; **b** IGF-1 protein levels determined by Western blot analysis

To validate the tissue distribution of pmIGF-1 expression, total RNA extracted from seven tissues (Sk muscle, heart, liver, lung, kidney, testis, and Se vesicle) of F1 Tg and Wt mice was analyzed by qRT-PCR. pmIGF-1 was expressed in Sk muscle, heart, liver, and testis of both Tg^+^ and Wt mice. Among the tissues tested, the expression level in Sk muscle was the highest. The expression in Sk muscle of Tg mice was 15 times higher than that in Sk muscle of Wt mice. The expression in heart or liver was not significantly different between Tg and Wt mice. Although the expression level in testes of Tg mice was much higher than that in testis of Wt mice, the expression levels were generally very low in testis (Fig. 4a). The seven tissue lysates of Tg mice were analyzed by Western blotting using a polyclonal human IGF-1 antibody with aa sequences the same as those of pmIGF-1 antibody, and four aa difference with mouse IGF-1 antibody. Among the analyzed tissues, pmIGF-1 could only be detected in Sk muscle and heart, and the expression level in Sk muscle was much higher than that in heart. The human mIGF-1 antibody specifically recognized Sk muscle IGF-1 with little or no cross-reactivity to other tissues (Fig. 4b). These results suggested that pmIGF-1 was specifically expressed in Sk muscle of Tg mice.

**Fig. 5:**
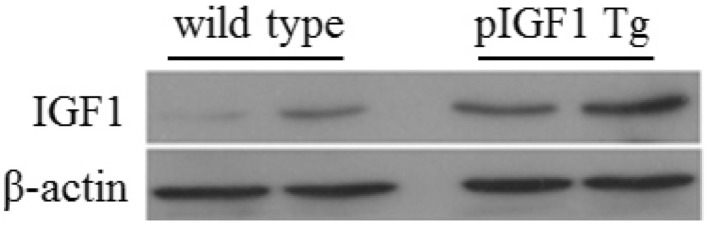
IGF-1 levels of gastrocnemius from Wt and pmIGF-1 Tg mice by Western blotting

The pmIGF-1 expression levels of Sk muscle from Tg mice were compared with those of Wt mice by the signal intensity on the Western blot determined by image analysis. The IGF-1 expression level in Tg mouse leg gastrocnemius was 3.5 times higher than that in Wt mice (Fig. 5).

### Growth Curve of Tg^+^ Mice

**Fig. 6:**
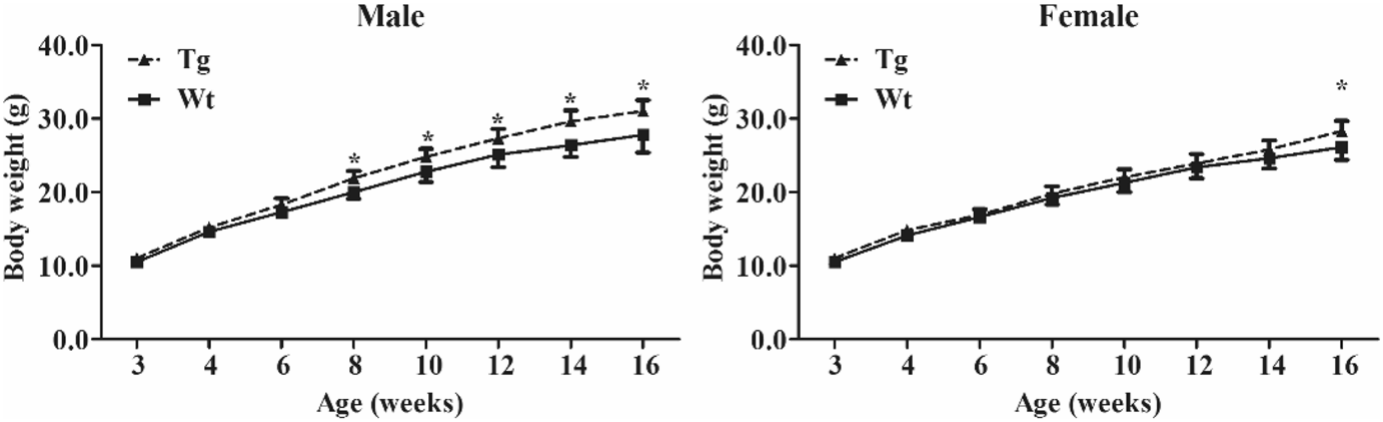
Growth curves of transgenic mice. *Tg* transgenic mice, *Wt* wild-type mice. *P < 0.05 (determined using the Student’s *t* test, comparing Wt and Tg mice)

The body weights of F1 Tg mice were determined from 3 to 16 weeks of age (Fig. 6). The body weight of male Tg mice was significantly heavier than that of Wt mice from 8 weeks old, and that of female Tg mice became heavier than that of Wt mice from 16 weeks of age (Fig. 6). These data suggested that exogenous IGF-1 expressed in Sk muscle facilitated the muscle growth of Tg mice at adult stage, and male mouse growth was affected at an earlier developmental stage than female.

### Skeletal Muscle Histology

**Fig. 7:**
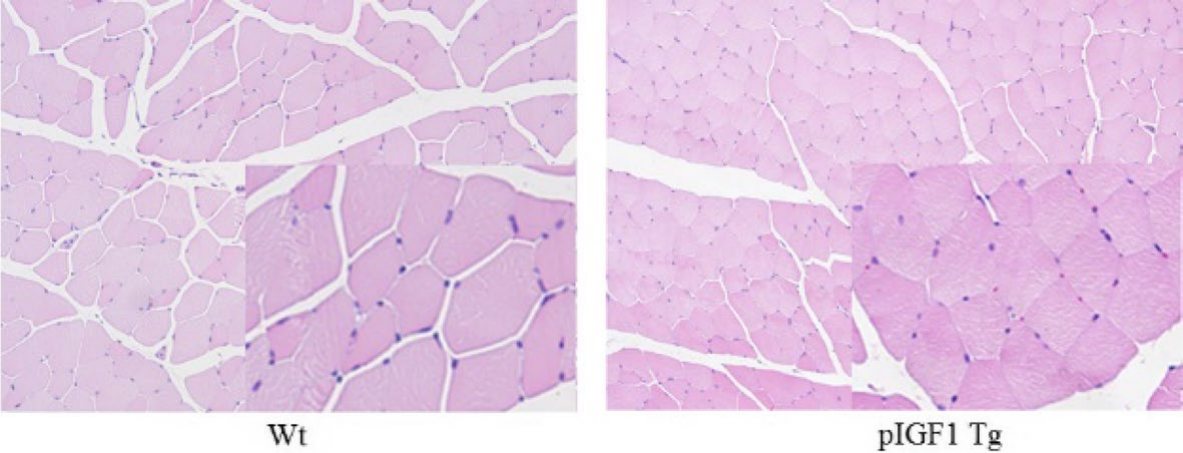
Hematoxylin and eosin histology of 16-week-old Tg and Wt mouse gastrocnemius. Images revealed the fiber hypertrophy in pmIGF-1 Tg. The magnification was ×100 and ×400 (inset)

To investigate the changes in muscle tissues, paraffin-embedded sections of 16-week-old mouse gastrocnemius were analyzed by H&E dye. The per unit numbers of myofibers from Tg mice were increased when compared with age-matched controls. The pmIGF-1 overexpression promoted myofiber proliferation and muscle hypertrophy (Fig. 7).

## Discussion

Alternative splicing of IGF-1 transcripts results in complexity of IGF-1 isoforms. The predominant isoform in Sk muscle is Class 1 (initiated at exon 1) IGF-1 Ea (Ea peptide from an exon 4–6 spliced variant). mRNA encoding the Class 1 IGF-1 Ea isoform is expressed locally in muscle (Musaro et al. 2001). To study the function of IGF-1 Ea, Tg mice expressing human mIGF-1 Ea or mIGF-1 Ea specifically in Sk muscle were developed. The overexpression of IGF-1 Ea can promote myofiber proliferation or heart hypertrophy (Coleman et al. 1995; Musaro et al. 2001). In this study, we constructed a myogenic vector expressing pmIGF-1 cDNA driven by pig skeletal α-actin gene 5′- and 3′- regulators. Although some studies indicated that skeletal α-actin was activated only in differentiated myoblasts (Sk and cardiac muscle cells) (Barton et al. 2002; Gunning et al. 1987; Musaro et al. 2001) and that the transgene is expressed only by differentiated muscle cells, our results showed that pig skeletal α-actin could strongly drive EGFP reporter gene expression in mouse C2C12 cells. One reason might be due to the SV40 enhancer included in the vector. The SV40 enhancer is known to be active in a wide variety of tissues and species. It contains a number of sequence motifs that can be bound by protein factors. The SV40 enhancer might have been responsible for activation of the skeletal α-actin promoter in C2C12 cells. In this paper, skeletal α-actin could drive EGFP expression in PEF cells, but not in PK15 cells, because the embryo fibroblast cells (PEF) might contain myofibroblast (Sassoon et al. 1988) and PK15 are pig kidney epithelial cells. In addition, the presence of the constructed vector led to EGFP expression at higher levels than those obtained with pEGFP-N1, which suggested that pig skeletal α-actin gene 5′- and 3′- regulators are strong in directing expression of the transgene.

Mouse transgenic studies indicated that human IGF-1 was expressed widely, which resulted in internal organ hyperplasia, such as heart, liver, and other organs, and that Tg mice did not live normally (Mathews et al. 1988). In this report, a transgenic mouse model expressing pmIGF-1 was developed by SB transposon integration. Western blotting analysis identified that pmIGF-1 was specifically expressed in Sk muscle from Tg mice, and other tissues showed no expression of pmIGF-1 except for low-level expression in cardiac muscle. These data suggest that we have succeeded in generating a transgenic mouse model to express pmIGF-1 peptide specifically in Sk muscle. G_0_ Tg mice lived normally to generate the next generation, which will aid in further study of the regulator mechanism of mIGF-1 in muscle development and transgene stability.

Studies reported high levels of hIGF-1 in gastrocnemius muscles of 6–9-month-old transgenic male mice and showed no correlation between gastrocnemius mass and levels of hmIGF-1 protein in the muscle, which suggested that there was a threshold for mIGF-1 levels above which no further enhancement of muscle growth occurred (Criswell et al. 1998). In this report, the overexpression of mIGF-1 led to significantly increased body weights of F1 adult mice (> 8 weeks old). For young mice (< 8 weeks old), endogenous mIGF-1 content in Sk muscle was high enough to promote muscle growth and transgenic mIGF-1 had no effect on mice. For adult mice (> 8 weeks old), endogenous mIGF-1 content decreased and exogenous mIGF-1 started to have an effect on muscle development. We also found that the effect of mIGF-1 overexpression on mice body weights was gender-specific. It is, perhaps, due to the different physiological mechanisms between male and female mice, such as growth hormone level or IGF-1 receptors level. Therefore, further research should be taken, for example, the expression level of IGF-1 or relative hormone at each development stage for both male and female mice. The mIGF-1 in muscles could combine with the IGF-1 receptors of adjacent cell surfaces through the paracrine system to active tyrosinase, which could regulate the expression of myogenic factors and then activate the proliferation and differentiation of muscle satellite cells and induce fusion of myocytes (Izumiya et al. 2008; Blaauw et al. 2009). Our histologic examination of gastrocnemius from 16-week-old male Tg mice also showed that pmIGF-1 overexpression in Sk muscle obviously increased the numbers of myofibers. Based on this study, further research should be undertaken on older Tg mice to obtain more information on the effect of local mIGF-1 expression in Sk muscle on muscle development.

Establishing an efficient gene integration method using nonviral vectors is important for creating transgenic mouse models. The total transgenic efficiency was only 2–5% (Sumiyama et al. 2010) when using the common method of pronuclear microinjection of plasmid DNA into fertilized eggs, because survival of injected embryos and integration of plasmid DNA are not efficient. To overcome these problems, novel transgenesis methods based on transposons have been developed in recent years. The foreign DNA was transposed from the plasmid to the genome and transmitted to the next generation very efficiently, with overall transgenic efficiency reaching > 20% (Sumiyama et al. 2010). In this paper, we obtained a 30% positive rate of transgenic founder mice by coinjection of the SB transposon vector together with transposase DNA into the pronucleus of fertilized eggs. Three transgenic lines were established by mating three male founder Tg mice with Wt C57BL/J6. The three male founders were all germ-line transgene mice, and the positive rate of F1 was about 90%. In conclusion, the SB transposon system is a simple and highly efficient transgenesis tool in mice. The high percentage of positive transgenic animals in F1 generation may be due to multi-site insertion mediated with SB transposon technique. However, that transposon mediates multi-site insert and multi-copy transgenes, which may cause high phenotypic variance and, sometimes, adverse genetic effects, is still a problem. It will take time to generate single copy transgene offspring.

In summary, we succeeded in effectively generating a Tg mice model strongly expressing pmIGF-1 specifically in Sk muscle. The mIGF-1 overexpression had an obvious effect on adult mouse muscle growth, which will provide good material for studying the molecular mechanism of muscle growth and development. Experimental data obtained from transgenic mouse models characterized by muscle-specific overexpression of IGF-1 suggested mIGF-1 as therapeutic agents in human muscular dystrophies (Fiorotto et al. 2003; Musarò et al. 2004; Shavlakadze et al. 2004). Pig as an animal model is better than rodents for human comparison (Smink et al. 2002). In this study, we validated the functions of pmIGF-1 and the skeletal α-actin regulator. On this basis, we will generate mIGF-1 transgenic pig models using the SB transposon system and cytoplasmic microinjection to obtain more-concrete data for human clinical use.

## Conclusions

In this study, a myogenic specific expression vector expressing pig mIGF-1 was successfully constructed using the 5′- and 3′-regulatory regions of porcine skeletal α-actin gene. Transgenic mice models expressing pig mIGF-1 specially in skeletal muscle were generated by the mediation of SB transposon with high transgene efficiency. Pig mIGF-1 overexpression in skeletal muscle of mice promoted myofibers hypertrophy and muscle production, and increased the average body weight of adult mice. Based on this study, transgenic pigs for pig endogenous IGF1 gene may be generated.
